# In Vitro Characterization and In Vivo Efficacy Assessment in *Galleria mellonella* Larvae of Newly Isolated Bacteriophages against *Escherichia coli* K1

**DOI:** 10.3390/v13102005

**Published:** 2021-10-06

**Authors:** Céline Antoine, Fanny Laforêt, Bob Blasdel, Abdoulaye Fall, Jean-Noël Duprez, Jacques Mainil, Véronique Delcenserie, Damien Thiry

**Affiliations:** 1 Bacteriology Laboratory, Department of Infectious and Parasitic Diseases, FARAH and Faculty of Veterinary Medicine, University of Liège, 4000 Liège, Belgium; celine.antoine@uliege.be (C.A.); fanny.laforet@uliege.be (F.L.); Jean-Noel.Duprez@uliege.be (J.-N.D.); jg.mainil@uliege.be (J.M.); 2 Food Science Department, FARAH and Faculty of Veterinary Medicine, ULiège, 4000 Liège, Belgium; Veronique.Delcenserie@uliege.be; 3 Vésale Bioscience, Vésale Pharmaceutica, 5310 Noville-sur-Mehaigne, Belgium; bob.blasdel@vesalepharma.com; 4 Genalyse Partner SA, En Hayeneux 62, 4040 Herstal, Belgium; afa@genalyse.eu

**Keywords:** bacteriophage, phage therapy, *Escherichia coli* K1, *Galleria mellonella*

## Abstract

Extra-intestinal *Escherichia coli* express several virulence factors that increase their ability to colonize and survive in different localizations. The K1 capsular type is involved in several infections, including meningitis, urinary tract, and bloodstream infections. The aims of this work were to isolate, characterize, and assess the in vivo efficacy of phages targeting avian pathogenic *E. coli* (APEC) O18:K1, which shares many similarities with the human strains responsible for neonatal meningitis. Eleven phages were isolated against APEC O18:K1, and four of them presenting a narrow spectrum targeting *E. coli K1* strains were further studied. The newly isolated phages vB_EcoS_K1-ULINTec2 were similar to the *Siphoviridae* family, and vB_EcoP_K1-ULINTec4, vB_EcoP_K1-ULINTec6, and vB_EcoP_K1-ULINTec7 to the *Autographiviridae* family. They are capsular type (K1) dependent and present several advantages characteristic of lytic phages, such as a short adsorption time and latent period. vB_EcoP_K1-ULINTec7 is able to target both K1 and K5 strains. This study shows that these phages replicate efficiently, both in vitro and in vivo in the *Galleria mellonella* model. Phage treatment increases the larvae survival rates, even though none of the phages were able to eliminate the bacterial load.

## 1. Introduction

Extra-intestinal *Escherichia coli* (ExPEC) is a pathotype that encompasses a great diversity of strains, and in which antibiotic resistance increases over the years. ExPEC can be normal inhabitants of the gut microbiota but can also express several virulence factors that allow them to colonize and survive in different localizations [1]. Among these factors, capsular type K1 is involved in ExPEC infections such as neonatal meningitis, urinary tract, and bloodstream infections [2,3,4,5]. *E. coli* is a leading cause of neonatal early-onset sepsis and meningitis in newborns, with a high proportion (approximately 80%) of strains expressing K1 capsule and frequently associated to a limited number of serotypes: O1, O7, O16, O18, and O45 [5]. The polysaccharidic structure of the K1 capsule allows *E. coli* to escape phagocytosis and thus survive and multiply outside the intestinal tract, as observed in neonatal meningitis, where it constitutes a determining virulence factor for the bacterial survival in the serum and for passage through the blood–brain barrier [6,7]. The recommended treatment in neonatal meningitis is based on empiric administration of antibiotics (ampicillin and gentamicin) [8]. Resistance to ampicillin is quite common, and, moreover, extended-spectrum beta-lactamase and multi-drug resistant strains have been described in several studies [9,10,11,12].

The presence of K1 capsulated *E. coli* has been demonstrated in humans but also in animals such as poultry, cows, and dogs, with several studies highlighting their zoonotic potential due to strong genetic and pathogenic similarities [13,14,15,16,17]. Avian pathogenic *E. coli* (APEC) isolates such as ST95 and ST131 or the O1, O2, and O18 serogroups might induce human extra-intestinal infections, although further investigation is needed to provide clear evidence of their zoonotic transmission to humans [18,19]. APEC isolates have the ability to produce clinical signs associated with human infections (sepsis, meningitis, and urinary tract infections) in rodents models and conversely, human strains causing neonatal meningitis are capable of inducing colibacillosis in poultry [19,20,21]. In addition, studies have shown that avian-associated plasmids ColV specific to APEC strains were also found in human isolates, again reinforcing the hypothesis of the zoonotic potential of APEC [19,22,23]. Antibiotic resistance in APEC is of concern and has been observed for all classes of antibiotics, except carbapenem; with prevalent resistances against ampicillin, tetracycline, trimethoprim, sulfamethoxazole, and streptomycin [18].

The antibiotic resistance of pathogenic bacteria is a real threat worldwide, in both humans and animals, and alternatives such as phage therapy have therefore been proposed to treat various infections. Phage therapy is not a new concept and was applied at the beginning of the 20th century after the discovery of bacteriophages in 1915 by Frederick Twort and in 1917 by Felix d’Herelle. However, the technique was rapidly supplanted with the advent of antibiotics outside of the former Soviet Union and allied states. With the emergence of multidrug resistant (MDR) bacteria and the need to find alternatives to antibiotics, phage therapy has gained renewed attention in western countries [24]. Indeed, phages present many advantages: they only infect bacterial cells and are highly specific; they have advantageous pharmacokinetics, due to self-replication at the infection site; and they have the ability to adapt to the development of resistance in targeted bacteria [24]. Nevertheless, phage-resistance has been demonstrated in studies assessing phage therapy, both in vitro and in vivo [25]. Several phages targeting *E. coli* K1 have been isolated and characterized since 1977 [26,27,28,29]. Considering their potential for phage therapy for treatment of diseases associated with *E.coli* K1, these phages were assessed in animal models and showed in vivo efficacy [27,29,30]. The applied phage treatments produced better results when using phages specifically targeting the K1 encapsulated *E. coli,* rather than non-specific phages [27,29,30].

Animal models are widely used in research on infectious diseases, the most widely used being vertebrates. The in vivo *Galleria mellonella* larvae (also called Greater wax moth larva) model has received attention given its low cost, ease of implementation, and being more ethically acceptable [31,32,33]. This model has the main advantage of having an innate immune system quite similar to humans, with humoral and cellular components, but it lacks an acquired immune system [34,35,36]. Several publications have already shown the advantages of this model in the preliminary evaluation of phages targeting antibiotic resistant bacteria in both humans and animals [37,38,39,40]. Moreover, one study investigating the use of bacteriophages targeting *E. coli* in *G. mellonella* larvae showed effectiveness with this model [41].

The aim of this work was to isolate and assess phages targeting avian pathogenic *E. coli* (APEC) O18:K1, a strain that shares many similarities with neonatal meningitis *E. coli* (NMEC) O18:K1. These phages were sequenced and characterized in vitro, and their potential in phage therapy was assessed in vivo in the *G. mellonella* larvae model.

## 2. Materials and Methods

### 2.1. Bacterial Strains

Ten APEC isolated from poultry in Belgium, France, and Spain between 1995 and 1999 were used. These isolates were responsible for clinical signs of colibacillosis and produced a lethality of 100% in 1-day-old chickens [14]. An *E. coli* serogroup collection of 179 strains previously characterized by seroagglutination was used for the host range, as well as Uropathogenic *E. coli* (UPEC), Enterohemorrhagic *E. coli* (EHEC), and Enteropathogenic *E. coli* (EPEC) strains (Tables S1 and S2). The capsular type K1 (*neuC*) was determined by PCR for all strains and capsular type K5 (*kfib*) for 3 strains (O10, O12, and UPEC 151.1). APEC isolates were also characterized by PCR to confirm their serogroup (O18) and the presence of the virulence gene *ibeA* involved in the passage of the blood–brain barrier. All bacteria were grown in LB Lennox broth at 37 °C. All primers used for bacterial characterization are listed in Table S3 and PCR results in Tables S1 and S2.

### 2.2. Phage Isolation and Propagation

**Figure 1:**
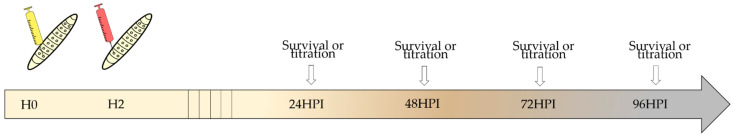
Representation of the survival and titration experiments of *Galleria mellonella* larvae. Larvae were inoculated at 2-h intervals. Survival assessment and phage: bacterial titrations were performed every 24 h for 96 h. HPI: hours post inoculation.

Wastewater samples used for isolation were collected in 2020 in wastewater treatment plants in Liège and Brussels and from one hospital (Ghent University Hospital). APEC 45 was selected for isolation and propagation. Phages were isolated by the enrichment method [42]. The waste water samples were centrifuged for 10 min at 9500× *g* and filtered at 0.2 µm. Then, the filtrate was added to an equivalent volume of LB Lennox (1 mM CaCl_2_, 1 mM MgSO_4_, Sigma-Aldrich, Saint-Louis, MO, USA) concentrated twice (LB2X), as well as 100 µL of bacterial culture at an optical density (OD) between 0.2 and 0.3 (~10^8^ CFU/mL). A sterility control, aiming to check the sterility of the filtrated water and a growth control were included. All tubes were incubated for 6 h at 37 °C until lysis was observed (Figure 1). The lysis tube and the growth control were then centrifuged at 9500× *g* and filtered at 0.2 μm. Between 2 and 20 μL of the 2 filtrates, as well as a 10× dilution of the lysis tube in TN buffer (Sigma-Aldrich, Saint-Louis, MO, USA), were spread on LB Lennox agar, covered with a bacterial overlay (OD: 0.2–0.3), and incubated at 37 °C until lysis plaques were visualized. These plaques were subcultured on LB Lennox agar 3 times. After plaque purification, lysates were made with 100 μL of the isolated phage and 20 μL of bacterial culture (OD: 0.2–0.3) in 5 mL of LB Lennox (1 mM CaCl_2_, 1 mM MgSO_4_). After centrifugation and filtration, the lysates were stored at 4 °C, −20 °C, and −80 °C. The phages were propagated by adding 20 mL of a new lysate to 400 mL of bacterial culture (OD: 0.2–0.3). After overnight incubation at 37 °C, 20 mL of chloroform was added to the culture and incubated for 10 min at 37 °C to release intracellular phages. The supernatant was then centrifuged for 1 h at 5000× *g* and filtered using a filtration unit (Nalgene, Thermo Fischer Scientific, Waltham, MA, USA) in which two filters of 0.45 µm and 0.2 µm (Whatman, GE healthcare, Maidstone, UK) were superimposed. The filtrates were then stored at 4 °C.

### 2.3. Host Range and Efficiency of Plating

The host ranges were performed using 4 µL spot tests in triplicate on LB Lennox agar (1 mM CaCl_2_, 1 mM MgSO_4_) covered with a bacterial overlay (OD: 0.2–0.3). Plates were then incubated at 37 °C overnight. The level of lysis was assessed using the scoring: clear lysis; opaque lysis; highly opaque lysis; non confluent plaques; and no lysis. Eleven phages were tested against a collection of 201 *E. coli* strains (Tables S1 and S2). For the efficiency of plating (EoP), 20 μL of each phage was serially diluted in 180 μL of PBS in a 96-well plate in triplicate. Drops of the dilutions (2 µL) were plated on an LB Lennox agar (1 mM CaCl_2_, 1 mM MgSO_4_) covered with a bacterial overlay (OD: 0.2–0.3). Plates were then incubated at 37 °C overnight. In order to obtain efficiency of plating, the titers on the tested strains were divided by the titer obtained on the propagation strain APEC 45.

### 2.4. Transmission Electron Microscopy

The purification method used was cesium chloride (CsCl) gradient ultracentrifugation followed by dialysis. Four densities were obtained by diluting CsCl (Cesium chloride ReagentPlus^®^ 99.9%, Sigma-Aldrich, Saint-Louis, MO, USA) in phage buffer (10 mM Tris, 10 mM MgSO_4_, 150 mM NaCl, Sigma-Aldrich, Saint-Louis, MO, USA): 1.33 g/cm^3^, 1.45 g/cm^3^, 1.5 g/cm^3^, and 1.7 g/cm^3^. A volume of each density was added successively, from the lowest to the highest density, in an ultracentrifuge tube (Ultra clear tubes 344058, Beckmann, Brea, CA, USA) using a Pasteur pipette. CsCl was added to a suspension of phage at a concentration of 10^10^ plaque-forming units (PFU)/mL and deposited on top of the gradient. The tube was then ultracentrifuged at 133,900× *g* for 3 h at 4 °C. The bands of purified and concentrated phages were collected and dialyzed at 4 °C with dialysis cassettes, following the manufacturer’s instructions (Slide-A-Lyzer ™ G2 Dialysis Cassettes, 10K MWCO, 3 mL, Thermo Fischer Scientific, Waltham, MA, USA). The dialyzed phages were stored at 4 °C.

The purified phages were negatively stained and analyzed by transmission electron microscopy (TEM) by the Electron Microscopy unit (Sciensano, Bruxelles, Belgique), as described by Mast and Demeestere, 2009 [43]. The grid was pre-treated with Alcian blue to increase hydrophilicity. The sample was placed on the grid using the grid-on-drop method. The grids were washed five times with distilled water and stained with 2% uranyl acetate (Agar Scientific, Standsted, UK). The TEM specimens were examined using a Tecnai Spirit microscope (FEI, Eindhoven, The Netherlands) operating at 120 kV.

### 2.5. DNA Sequencing and Phage Genome Annotation

DNA of purified phages was extracted using a DNA extraction kit MagCore^®^ Viral Nucleic Acid Kit with 60 μL elution volume (Atrida, NL), DNeasy Blood & Tissue kit (Qiagen, Crawley, UK) following the manufacturer’s instructions. Sequencing libraries were constructed using an Illumina Nextera XT DNA sample preparation kit and subsequently sequenced on an Illumina MiSeq instrument (Illumina, San Diego, CA, USA).

The phage genomes were assembled into single contigs using CLC Genomics Workbench v21.0.4 (using default parameters, https://digitalinsights.qiagen.com/, accessed on 31 August 2021). CDS calls were initially made based on analyses with RAST [44], while protein functions predicted by RAST were then reanalyzed using searches for conserved domains with InterPro v86.0 [45] and HHpred v57c8707149031cc9f8edceba362c71a3762bdbf8 [46]. Closely related phages deposited in GenBank were identified using BLASTn analysis. Figures comparing phage genomes were generated using Easyfig v2.2.5 [47].

### 2.6. Adsorption Time and Low MOI Phage Kinetics

The adsorption time and low MOI phage kinetics were performed following Merabishvili et al. [48], with adaptations. For the adsorption time, each phage (20 µL) was mixed with 5 mL of APEC 45 (OD: 0.25) at a multiplicity of infection (MOI) of 0.001 and incubated at 37 °C. Samples of 500 µL were removed after 3, 5, 8, 10, and 15 min and diluted 10 times in 4 mL LB broth and 0.5 mL chloroform. After 30 min of incubation at room temperature, the number of unadsorbed phages was titrated in triplicate. The adsorption time was determined as the time when the ratio of non-adsorbed phage over the initial phage number was under 0.1. For the low MOI phage kinetic experiment, 1 mL of 10^8^ CFU/mL of APEC 45 was exposed to each phage at a MOI of 0.001. The adsorption process was allowed to occur for 8 min at 37 °C. The culture was centrifuged at 13,000× *g* for 1 min, resuspended in 10 mL of LB Lennox and incubated for 100 min at 37 °C. Samples were removed every 5 min and titrated in triplicate. The latent period was the time between phage adsorption and the beginning of phage release.

### 2.7. Temperature and pH Stability

Temperature stability was assessed by incubating 1 mL of 10^8^ PFU/mL phage samples at different temperatures (25 °C, 37 °C, 45 °C, and 60 °C). For pH stability, 100 µL of 10^9^ PFU/mL phage samples were diluted in 900 µL of PBS at different pH (2, 4, 6, 8, 10, and 12). All samples were incubated for 1 h and subsequently titrated in biological triplicate. Long term stability was evaluated by titrating before and after 8 months of storage at 4 °C, −20 °C, and −80 °C. For conservation, glycerol (15% *v/v*, Fisher chemical, Pittsburgh, PA, USA) was added to phage lysates, according to González-Menéndez et al. [49].

### 2.8. In Vivo Assay: Galleria Mellonella

To determine the optimal inoculation dose, 6 groups of 10 *G. mellonella* larvae (Nusect, Deerlijk, Belgium) were inoculated using an automatic injector (Cole Parmer, Vernon Hills, IL, USA) with 10 µL of APEC 134 at 6 different concentrations, ranging from 10 CFU/10 µL to 10^6^ CFU/10 µL. Each larva was inoculated in the last left proleg with a BD Plastipak™ 1 mL sterile syringe (Becton-Dickinson, Franklin Lakes, NJ, USA) and a sterile 30-gauge needle (Terumo corporation, Tokyo, Japan). The optimal inoculation dose was expected to cause a lethality of between 90 and 100% after 4 days.

Four experiments were carried out in order to evaluate the individual efficacy of 4 phages (vB_EcoS_K1-ULINTec2, vB_EcoP_K1-ULINTec4, vB_EcoP_K1-ULINTec6 and vB_EcoP_K1-ULINTec7) on the survival of *G. mellonella* larvae. For each experiment, 150 larvae were divided into 5 groups (Table 1). Each larva was inoculated using an automatic injector (Cole Parmer, Vernon Hills, IL, USA) in the last left proleg for the first injection followed by a second injection 2 h later in the last right proleg. Injections were performed using BD Plastipak™ 1 mL sterile syringes (Becton-Dickinson, Franklin Lakes, NJ, USA) and sterile 30-gauge needles (Terumo corporation, Tokyo, Japan). The larvae were incubated at 37 °C and mortality was evaluated every 24 h (Figure 1). Kaplan-Meier survival curves were generated to assess the survival of the different groups using R-commander (Rcmdr v2.6-0) [50]. Logrank tests were performed to highlight any significant difference in survival rates between the groups (*p* ≤ 0.05).

**Figure 2:**
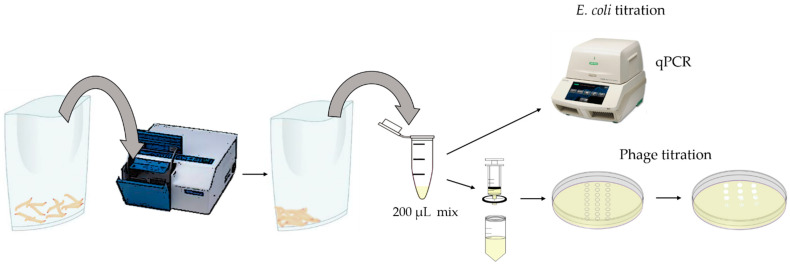
Representation of the titration process of bacteria and phages contained in the *G. mellonella* larvae.

To assess bacterial and phage titer evolutions, 600 larvae were divided into 5 groups. They were inoculated following the same protocol used for the survival experiment. The titrations of APEC 134 and phages were performed every 24 h for 96 h in biological triplicate, with larvae grinded in a homogenizer (Stomacher) in groups of 10 individuals. The mix (200 µL) was immediately diluted in PBS and weighed. Half of the sample was filtered with a 0.2 µm sterile syringe filter (ref.514-0073, VWR, Leicestershire, UK). The filtrate was titrated in triplicate with 10-fold serial dilutions and plated on LB Lennox agar (1 mM CaCl_2_, 1 mM MgSO_4_) with a bacterial overlay (OD: 0.2–0.3). Plates were incubated at 37 °C for 16 to 18 h before counting. For APEC 134 titrations, samples were pre-treated with DNaseI (1 mg/mL, Sigma-Aldrich, Saint-Louis, MO, USA) and RNaseA (100 mg/mL, Sigma-Aldrich, Saint-Louis, MO, USA). DNA was then extracted using a DNeasy^®^ Blood & Tissue kit (Qiagen, Hilden, Germany), following the manufacturer instructions. Bacterial titrations were performed using a qPCR targeting the *neuB* virulence gene using the primer pair neuB-F: 5′-ATGTTTCAGTTCATCAGGTTCTATTGA-3′ and neuB-R: 5′-TCTGATCATTCTAGCGGGTTTTTATG-3′ and probe neuBK1: 5′-[6FAM]-TTATTCCATAAGGCACCGCCGCAA-[BHQ1]-3′ [51]. Real-time PCR assays were performed with a CFX96 Touch Real-Time PCR Detection System (Biorad, Hercules, CA, USA), according to the manufacturer’s recommendations in 20 µL reaction volume. The enzyme used was the FastGene^®^ Probe 2 × qPCR Universal Mix (Nippon genetics, Tokyo, Japan). The primers and probe (Eurogentec, Seraing, Belgium) were used at a final concentration of 400 nM and 200 nm, respectively. The following steps were applied: enzyme activation at 95 °C for 3 min followed by 40 cycles of denaturation at 95 °C for 5 s and annealing at 60 °C for 30 s. All negatives samples were also checked by qPCR using the universal prokaryotes (16 S rRNA) primer pair (500 nM final concentration) 926F: 5′-AAACTCAAAKGAATTGACGG and 1062R: 5′-CTCACRRCACGAGCTGAC-3′ [52] and the enzyme Takyon™ No ROX SYBR 2X MasterMix blue dTTP (Eurogentec, Seraing, Belgium). The following steps were applied: enzyme activation at 95 °C for 5 min, followed by 35 cycles of denaturation at 95 °C for 15 s, annealing at 61.5 °C for 15 s, elongation at 72 °C for 30 s. The phage and bacterial processes are illustrated in Figure 2. Kruskall–Wallis and Mann–Whitney tests were performed using R-commander (Rcmdr v2.6-0) [50] to statistically assess the efficacy of the phages.

## 3. Results

### 3.1. Phage Isolation

Eleven phages were isolated against APEC 45. All were propagated and were able to reach high titers (>10^9^ PFU/mL) in liquid culture. Four phages specific of the K1 capsular type (K1-dependent) were named according to the nomenclature and studied more extensively: vB_EcoS_K1-ULINTec2, vB_EcoP_K1-ULINTec4, vB_EcoP_K1-ULINTec6, and vB_EcoP_K1-ULINTec7.

### 3.2. Host Range and Efficiency of Plating

**Figure 3:**
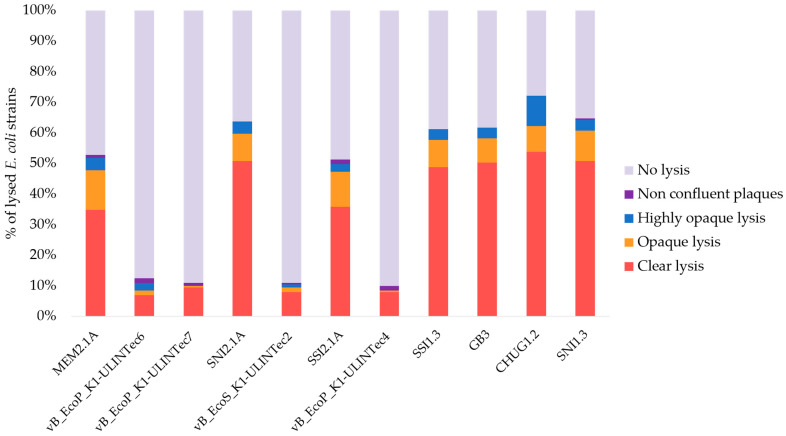
Percentage of lysed strains and degree of lysis of the 11 phages isolated against *E. coli* O18:K1.

The host range was evaluated for all phages isolated against *E. coli* O18:K1 (APEC 45). The results are summarized in Figure 3. Four phages showed a narrow spectrum (vB_EcoS_K1-ULINTec2, vB_EcoP_K1-ULINTec4, vB_EcoP_K1-ULINTec6, and vB_EcoP_K1-ULINTec7) that includes O18:K1 and other K1 strains. Several serogroups, as well as two UPEC strains lysed by these four phages, were associated with the K1 capsular type, except serogroups O10, O12, and UPEC strain 151.1 that encode the *kfib* virulence gene associated with capsular type K5 (Table S1). These three strains were only lysed by vB_EcoP_K1-ULINTec7 and not by the three other narrow spectrum phages.

Since the host range spots tests tended to overestimate the virulence of the phages tested, EoP was performed for the four phages (vB_EcoS_K1-ULINTec2, vB_EcoP_K1-ULINTec4, vB_EcoP_K1-ULINTec6, and vB_EcoP_K1-ULINTec7) and suggested abortive infection (non-productive phage infection that kill bacteria) in some cases. No phage could effectively lyse all of the K1 strains tested. vB_EcoS_K1-ULINTec2 showed the narrowest spectrum but reached good EoP values on the lysed strains. vB_EcoP_K1-ULINTec7 have the largest spectrum for K1 strains and can lyse K5 strains. The results of the EoP are detailed in Table 2.

### 3.3. Transmission Electron Microscopy

**Figure 4:**
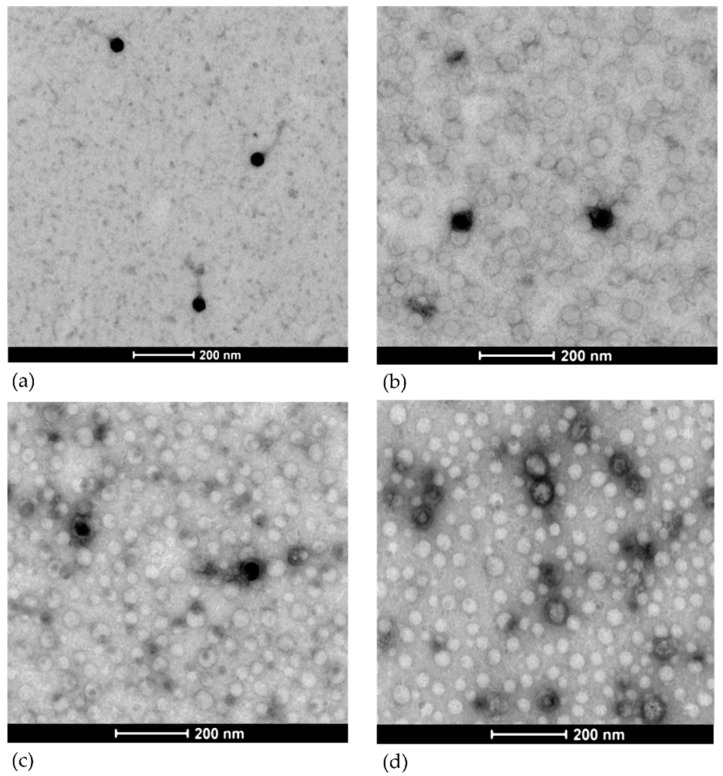
Negative staining transmission electron microscopic images of (**a**) vB_EcoS_K1-ULINTec2, *Siphoviridae*. (**b**) vB_EcoP_K1-ULINTec4, *Podoviridae*. (**c**) vB_EcoP_K1-ULINTec6, *Podoviridae*. (**d**) vB_EcoP_K1-ULINTec7, *Podoviridae*.

Electron microscopy analyses showed that the four phages can be assigned to the order of *Caudovirales*. vB_EcoS_K1-ULINTec2 is characterized by an icosahedral symmetric non-enveloped head, with a diameter of approximately 50 nm, and a flexible, non-contractile tail approximately 150 nm in length, and is attributed to the *Siphoviridae* family (Figure 4a). vB_EcoP_K1-ULINTec4 is characterized by an icosahedral symmetric non-enveloped head, with a diameter of approximately 50 nm, and a very short tail, and is attributed to the *Podoviridae* family (Figure 4b). vB_EcoP_K1-ULINTec6 is characterized by an icosahedral symmetric non-enveloped head, with a diameter of approximately 60 nm, and a very short tail, and is attributed to the *Podoviridae* family (Figure 4c). vB_EcoP_K1-ULINTec7 is characterized by an icosahedral symmetric non-enveloped head, with a diameter of approximately 55 nm, and a very short tail, and is attributed to the *Podoviridae* family (Figure 4d).

### 3.4. DNA Sequencing and Phage Genome Annotation

**Figure 5:**
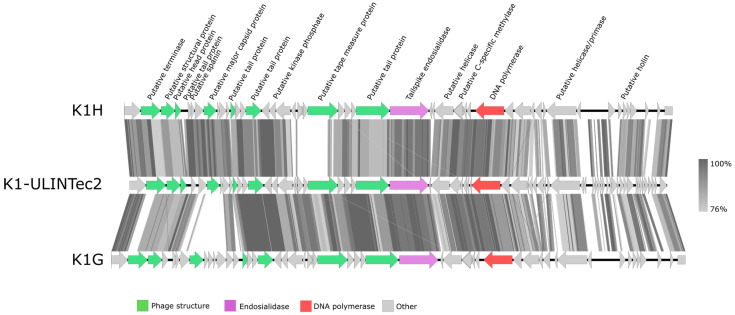
Comparative genomics of phages vB_EcoS_K1-ULINTec2 (K1-ULINTec2), K1H and K1G.

**Figure 6:**
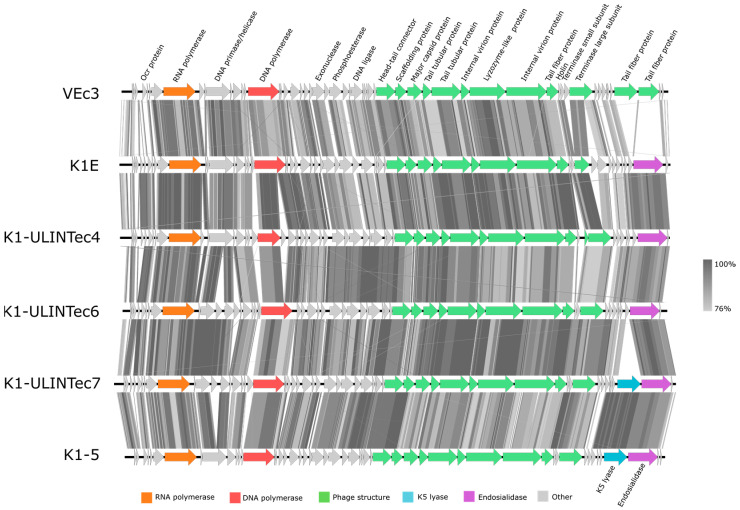
Comparative genomics of phages vB_EcoP_K1-ULINTec4 (K1-ULINTec4), vB_EcoP_K1-ULINTec6 (K1-ULINTec6), vB_EcoP_K1-ULINTec7 (K1-ULINTec7), and VEc3, K1E and K1-5.

Phage genome analysis showed that the four phages belong to the *Caudovirales* order. Phage vB_EcoS_K1-ULINTec2 (40,815 bp) is a siphovirus related to the *Kagunavirus* genus. It shares 96% and 94% identity with phages K1G and K1H, respectively. Phages vB_EcoP_K1-ULINTec4 (45,159 bp), vB_EcoP_K1-ULINTec6 (44,728 bp), and vB_EcoP_K1-ULINTec7 (46,142 bp) belong to the *Autographiviridae* family and to the *Vectrevirus* genus. K1E shares the strongest identity with vB_EcoP_K1-ULINTec4 (97%), followed by both vB_EcoP_K1-ULINTec6 and vB_EcoP_K1-ULINTec7 (94%). Phage VEc3 shares between 94 and 96% identity with the three *Vectrevirus,* and phage K1-5 showed 92% identity with vB_EcoP_K1-ULINTec7. Homology detection and structure prediction by HMM-HMM analysis HHpred [53] revealed that all phages encode a putative endosialidase, and that vB_EcoP_K1-ULINTec7 additionally encodes a K5 lyase not present in the three other phages isolated. No gene related to lysogeny or virulence was revealed, suggesting the strictly lytic characteristics of these phages. Comparisons of phage genomes are shown in Figure 5 (*Kagunavirus*) and Figure 6 (*Vectrevirus*). Sequencing was submitted as NCBI BioProject SUB10289878. GenBank accession numbers are MZ997838 (vB_EcoS_K1-ULINTec2), MZ997839 (vB_EcoP_K1-ULINTec4), MZ997840 (vB_EcoP_K1-ULINTec6), and MZ997841 (vB_EcoP_K1-ULINTec7).

### 3.5. Adsorption Time and Low MOI Phage Kinetics

**Figure 7:**
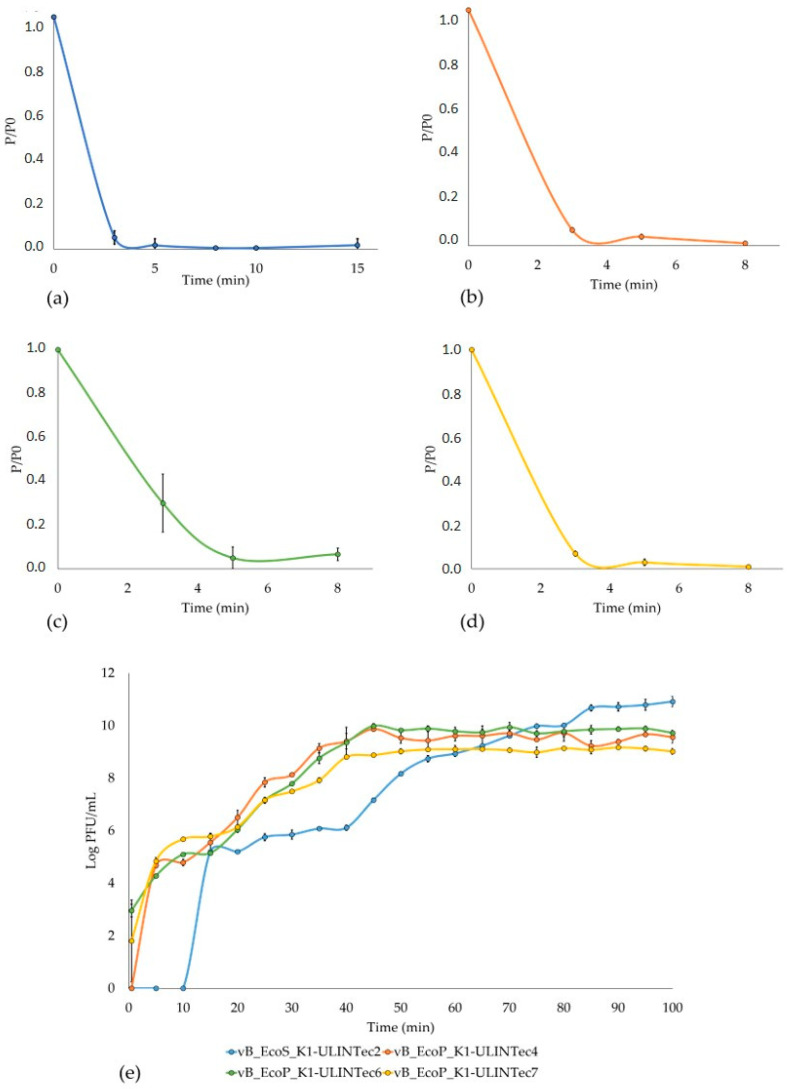
Adsorption time of (**a**) vB_EcoS_K1-ULINTec2, (**b**) vB_EcoP_K1-ULINTec4, (**c**) vB_EcoP_K1-ULINTec6, (**d**) vB_EcoP_K1-ULINTec7, and low MOI phage kinetics (**e**). P_0_: initial phage concentration. P: number of free phages. The results are the mean value of three titrations.

In order to obtain more information about the infection cycle of these phages, the adsorption time and the phage production at low MOI were assessed. The respective adsorption times of vB_EcoS_K1-ULINTec2, vB_EcoP_K1-ULINTec4, vB_EcoP_K1-ULINTec6, and vB_EcoP_K1-ULINTec7 were <3 min, <3 min, 5 min, and <3 min (Figure 7 a–d). The latency periods, respectively, lasted 10–15 min, <5 min, <5 min, and <5 min (Figure 7e). The highest phage production was reached in 80 min by vB_EcoS_K1-ULINTec2, with a titer of 6 × 10^10^ PFU/mL, followed by the three *Vectrevirus* that reached the stationary phase after 45 min. The respective phage productions of vB_EcoP_K1-ULINTec4, vB_EcoP_K1-ULINTec6, and vB_EcoP_K1-ULINTec7 were 3.7 × 10^9^ PFU/mL, 6.7 × 10^9^ PFU/mL, and 1.2 × 10^9^ PFU/mL (Figure 7e).

### 3.6. Temperature and pH Stability

**Figure 8:**
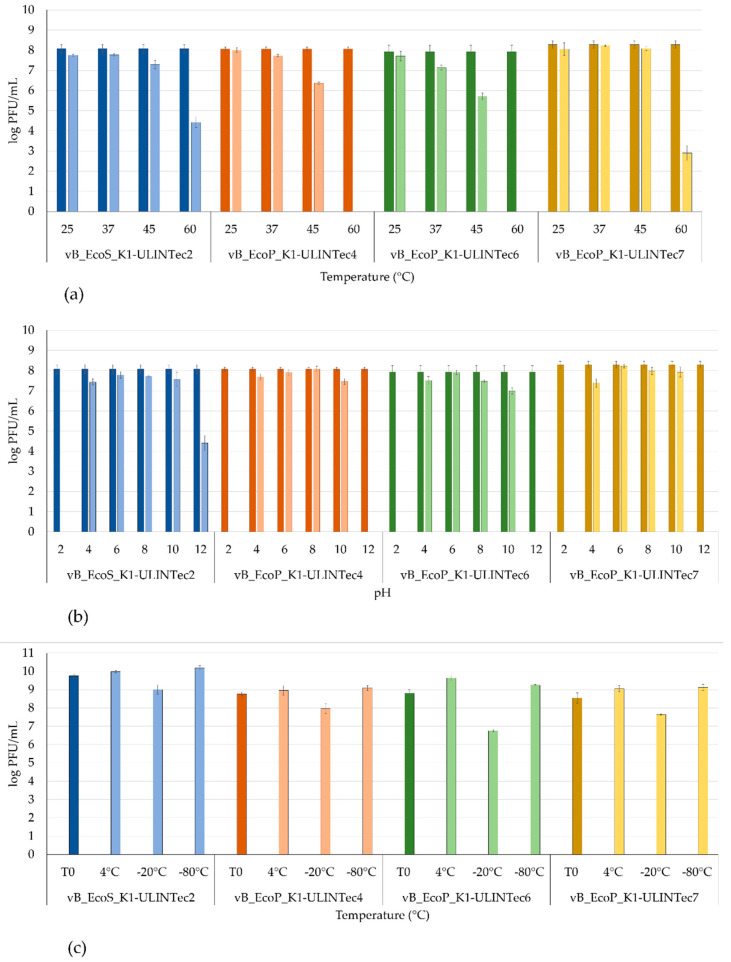
Temperature (**a**), pH (**b**), and storage (**c**) stability results of phages vB_EcoS_K1-ULINTec2, vB_EcoP_K1-ULINTec4, vB_EcoP_K1-ULINTec6, and vB_EcoP_K1-ULINTec7. The results are the mean value of three titrations. Dark bars represent the concentration measured before testing.

All phages remained stable (<1 log PFU/mL reduction) at temperatures of 25 °C and 37 °C, and phages vB_EcoS_K1-ULINTec2 and vB_EcoP_K1-ULINTec7 were also stable at 45 °C (Figure 8a). At 60 °C, phages vB_EcoP_K1-ULINTec4 and vB_EcoP_K1-ULINTec6 were not detectable, while the loss of titer of vB_EcoS_K1-ULINTec2 and vB_EcoP_K1-ULINTec7 was about 4 log PFU/mL and 5 log PFU/mL, respectively (Figure 8a). Regarding the pH stability, the four phages remained stable between pH 4 and pH 10, but none of them were detected at pH 2, and only vB_EcoS_K1-ULINTec2 was titrated at pH 12 with a loss of 4 log PFU/mL (Figure 8b). The 8 months storage at 4 °C and −80 °C allowed maintaining the titers of all phages, unlike the −20 °C storage, which led to a decrease in phage titer of approximatively 0.5 and 1.5 log PFU/mL (Figure 8c).

### 3.7. In Vivo Assay: Galleria Mellonella

**Figure 9:**
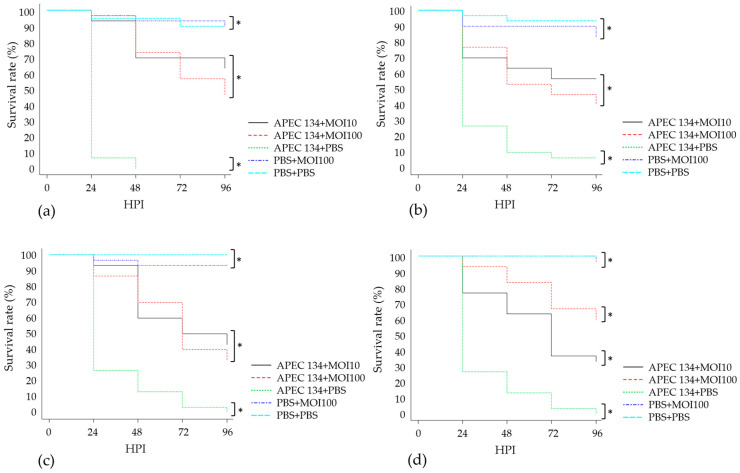
Kaplan–Meier survival curves of the experiments with *G. mellonella* larvae inoculated with *E. coli* O18:K1 (APEC 134) or PBS and treated with phages (**a**) vB_EcoS_K1-ULINTec2, (**b**) vB_EcoP_K1-ULINTec4, (**c**) vB_EcoP_K1-ULINTec6, (**d**) vB_EcoP_K1-ULINTec7, or PBS two hours later. Each group contained 30 larvae separated in 3 groups of 10 larvae. MOI: multiplicity of infection. HPI: hours post inoculation. *p*-value (*) < 0.05.

Groups treated with phage vB_EcoS_K1-ULINTec2 showed better survival rates than the infected untreated control (APEC 134 + PBS). The larvae survival rates of the phage treated groups were nevertheless lower than the uninfected controls (PBS + PBS and PBS + phage) (50–60% vs. 90% of survival 96 HPI). The larvae survival rates of the groups treated with a MOI of 10 or 100 were not significantly different. Quite similar results were obtained for the phages vB_EcoP_K1-ULINTec4 and vB_EcoP_K1-ULINTec6, for which an increased survival was observed after the phage treatment. Larvae treated with vB_EcoP_K1-ULINTec7 showed better survival rates than the infected untreated control (APEC 134 + PBS). The larvae survival rates of the groups treated with these phages were nevertheless lower than the uninfected controls. This phage allowed a better larvae survival rate in the group treated at a MOI of 100 (60% of survival), in comparison with a MOI of 10 (35% of survival). The Kaplan–Meier survival curves are shown in Figure 9.

**Figure 10:**
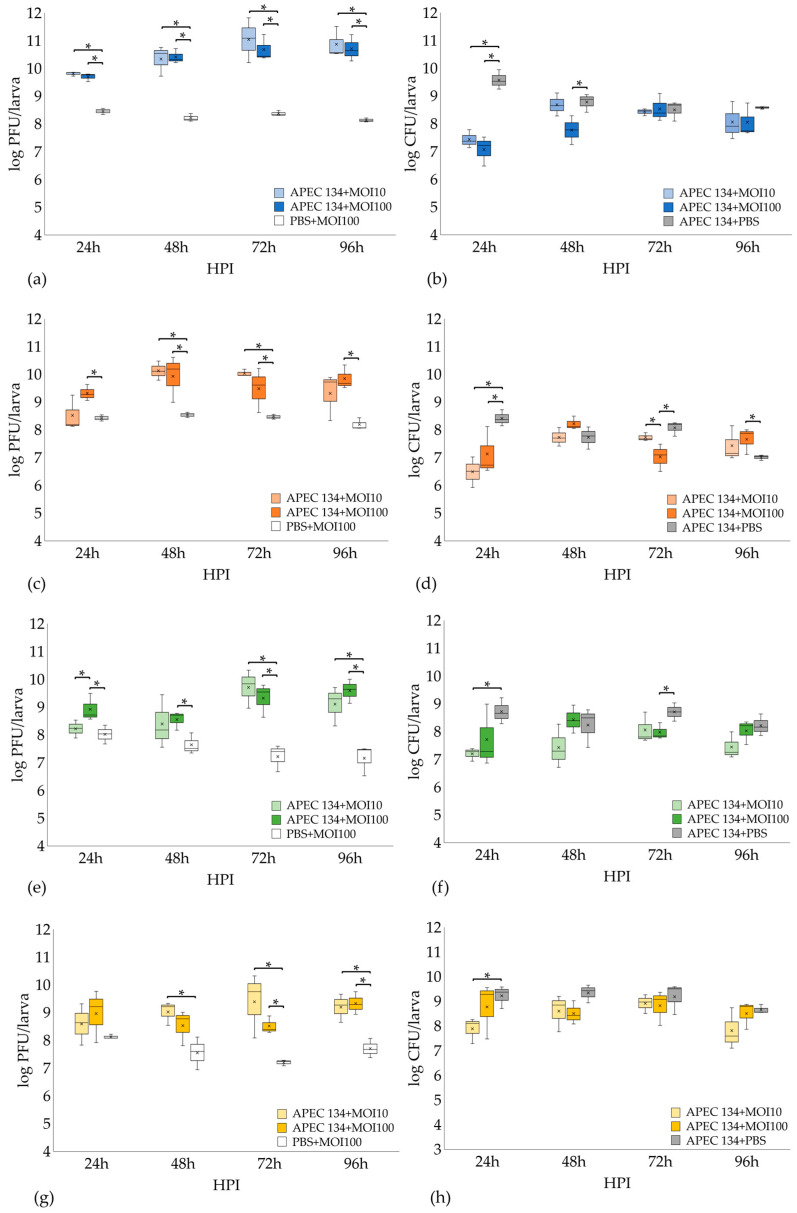
Phage vB_EcoS_K1-ULINTec2 (**a**), vB_EcoP_K1-ULINTec4 (**c**), vB_EcoP_K1-ULINTec6 (**e**), vB_EcoP_K1-ULINTec7 (**g**), and bacterial vB_EcoS_K1-ULINTec2 (**b**), vB_EcoP_K1-ULINTec4 (**d**), vB_EcoP_K1-ULINTec6 (**f**), and vB_EcoP_K1-ULINTec7 (**h**) concentrations measured during the titration experiments using the *G. mellonella* model. Titrations were performed every 24 h for 96 h. The results present data from 3 independent experiments. *p*-value (*) < 0.05. MOI: multiplicity of infection. HPI: hours post inoculation.

In order to deeply assess the efficacy of these phages, bacterial and phage concentrations were measured every 24 h for 96 h. In the vB_EcoS_K1-ULINTec2 experiment, phage titers significantly increased in the infected treated groups (MOI 10 and MOI 100) compared to the phage control group at each titration. In the phage control group, the dose inoculated at H0 remained stable over the 4 days (Figure 10a). After 24 HPI, bacterial concentrations were significantly lower in the treated infected groups (MOI 10 and 100) compared to the untreated infected control. At 48 HPI post inoculation, only the group treated with a MOI of 100 produced a lower concentration. Bacterial titers were not significantly different at 72 HPI and 96 HPI (Figure 10b).

Larvae treated with phage vB_EcoP_K1-ULINTec4 at a MOI of 100 showed significantly higher phage concentrations than the phage control group. The group treated with a MOI of 10 was not significantly different from the control but still showed an increase in titer compared to H0 (10^7^ PFU/larva). It was at 48 HPI that larvae treated with a MOI of 10 and 100 both become significantly higher than the phage control group. Phage titers were still significantly higher at both 72 HPI and 96 HPI in larvae treated with an MOI of 100, and only at 72 HPI with a MOI of 10. In the phage control group, the dose inoculated at H0 remained stable for the 4 days of titration (Figure 10c). Bacterial concentrations were significantly lower in the infected treated groups (MOI 10 and MOI 100); 24 HPI compared to the untreated infected control. After 48 HPI the bacterial titers were no longer significantly different from each other. At 72 HPI, larvae treated with a MOI of 100 showed lower bacterial concentrations than the MOI 10 and untreated infected groups. The bacterial titer of the MOI 100 treated group was significantly higher than the untreated group, at 96 HPI (Figure 10d).

Groups treated with vB_EcoP_K1-ULINTec6 with a MOI of 100 showed a concentration significantly higher than the groups treated with a MOI of 10 and higher than the phage control group at 24 HPI. At 48 HPI, 72 HPI, and 96 HPI, phage concentrations in larvae treated with a MOI of 100 were significantly higher than the phage control group, while larvae treated with a MOI of 10 led to significantly higher concentrations at 72 HPI and 96 HPI (Figure 10e). For bacterial titers, larvae treated with a MOI of 10 showed a significantly lower titer than the untreated infected control at 24 HPI. Groups treated with a MOI of 100 were significantly lower than the infected untreated groups (Figure 10f).

The titration experiment performed with vB_EcoP_K1-ULINTec7 showed that phage titers were not significantly different between the infected treated groups and the phage control group at 24 HPI. The phage concentration was significantly higher in the MOI 10 treated group compared to the phage control group at 48 HPI. At 72 HPI and 96 HPI the two infected treated groups (MOI 10 and MOI 100) showed significantly higher phage concentrations than the phage control group (Figure 10g). The bacterial concentration measured in the MOI 10 treated group was significantly lower than in the untreated group at 24-h post-inoculation, which was not the case for the MOI 100 treated group. At 48, 72, and 96 h post-inoculation, the bacterial titers were not significantly different between the groups (Figure 10h).

## 4. Discussion

Among the 11 phages isolated against the avian strain O18:K1 (APEC 45), four had a narrow host spectrum specific to the capsular type K1: vB_EcoS_K1-ULINTec2, vB_EcoP_K1-ULINTec4, vB_EcoP_K1-ULINTec6, and vB_EcoP_K1-ULINTec7. This specificity represents an advantage for phage therapy, as it allows a precise targeting of specific bacteria, in contrast to antibiotic treatments, which affect a wider range of species, thus disrupting the microbiota.

The efficiency of plating showed that the host spectrum of these phages was even narrower than observed for the host range. Some strains were very weakly, or not at all, lysed. Some lysis reactions showed that the phages were not able to replicate in these strains, but still produced lytic zones at the highest concentrations. This lysis may have been due to a phenomenon called abortive infection, which describes a non-productive phage infection that kills bacteria [54], or to the presence of an enzyme degrading the polysialic acid of the K1 capsule. Several phages described in the literature, indeed, possess endosialidases that allow them to degrade the K1 capsule of *E. coli* [27,55,56]. Phages able to infect *E. coli* K1 were divided into two categories, according to the essentiality of the presence of the K1 capsule for infection: K1-dependent (K1-dep) and K1-independent (K1-ind). The results obtained in vitro and in vivo demonstrated the superiority in terms of efficacy of K1-dep phages compared to K1-ind phages [27,30,55]. The phage genomic analysis of vB_EcoS_K1-ULINTec2, vB_EcoP_K1-ULINTec4, vB_EcoP_K1-ULINTec6, and vB_EcoP_K1-ULINTec7 showed that these phages appear to encode the same endosialidase activity, allowing them to target the *E. coli* K1 capsule. The host range and efficiency of plating experiments also showed that the phage vB_EcoP_K1-ULINTec7 was able to replicate, not only on K1 strains, but also on K5 strains. These results are consistent with the genomic analysis of this phage and with the existence of other phages encoding two types of enzymes, one degrading the K1 capsule (endosialidase) and the other degrading the K5 capsule (K5 lyase); these capsules being chemically different [28,57,58]. These enzymes are organized into a multiprotein complex attached via an adapter protein to the virus portal vertex, through which the DNA is ejected during infection [58].

At a morphological and taxonomic level, the electron microscopy images assigned the phage vB_EcoS_K1-ULINTec2 to the *Siphoviridae* family and the other three phages (vB_EcoP_K1-ULINTec4, vB_EcoP_K1-ULINTec6, and vB_EcoP_K1-ULINTec7) to the *Podoviridae* family. This was also the case for the K1-dep phages isolated in other publications [30,55,56]. The phage vB_EcoP_K1-ULINTec7 is a *Podoviridae,* as is the phage K1-5, which is also able to lyse both K1 and K5 strains [28,57]. At a genomic level, vB_EcoS_K1-ULINTec2 has significant similarity to the *Kagunavirus* genus (*Siphoviridae* family) and vB_EcoP_K1-ULINTec4, vB_EcoP_K1-ULINTec6, and vB_EcoP_K1-ULINTec7 each have similarity to the *Vectrevirus* genus (*Autographiviridae* family). *Autographiviridae* was previously a subfamily of *Podoviridae* but since 2019 has had a family of its own in the *Caudovirales* order.

The results observed during the in vitro kinetic experiments (adsorption time and low MOI phage kinetics) demonstrate the productive infection of these phages. The adsorption times and latent periods were very short, except for the vB_EcoS_K1-ULINTec2 phage, whose latent period was longer than for the other phages (10–15 min).

In vivo experiments with *G. mellonella* larvae showed increased survival rates in larvae treated with phages vB_EcoS_K1-ULINTec2, vB_EcoP_K1-ULINTec4, vB_EcoP_K1-ULINTec6, or vB_EcoP_K1-ULINTec7 at a MOI of 10 or 100. The survival rates of these larvae were not as good as the PBS and phage control groups, but this could be explained by the 2-h interval applied in the present study. Indeed, only a few hours after inoculation of APEC 134 into the larvae, some of them already showed visible signs of infection (decreased activity, melanization). A 1-h interval might have further increased survival, but the present experiment showed a significant effect despite the fast deterioration of the larvae.

Inoculated phages seem to replicate well in the model, as shown by the phage counts measured in the larvae over 96 h. Nevertheless, the bacterial counts were also rather high. Significant effects on these counts can be observed, mainly at 24 and 48 HPI. After this time, the phage-treated groups showed bacterial titers equivalent to the infected control, which led to a decrease in larvae survival after 24 h. One hypothesis could be that the bacteria detected by qPCR no longer express their K1 capsule, thus decreasing or suppressing phage replication, but also decreasing the virulence of the inoculated bacteria. This effect has already been observed by other researchers, who have shown that the use of endosialidase selectively degrades the PSA capsule on the surface of *E. coli* K1 strains, thus modifying its phenotype and reducing bacteremia and mortality in rats [59]. Moreover, qPCR bacterial titers may partially reflect the presence of dead cells, even if a DNase pre-treatment is applied. The bacterial dose used (10^6^ CFU/larva) resulted in a very high mortality in the 24 h following inoculation in the infected group. When determining the optimal inoculation dose, an inoculum of 10^5^ CFU/larva resulted in a mortality of about 30%. This highlights the acute aspect of infection by this strain with a concentration of 10^6^ CFU/larva. Other experiments conducted on mice showed that a dose of 10^8^ CFU/mouse injected intravenously resulted in 100% mortality within 24–48 HPI, while a dose of 10^7^ CFU/mouse had little impact on mortality [60]. The better survival of *G. mellonella* larvae could therefore also be attributed to a rapid and transient decrease in bacterial titer during the 24 h following inoculation.

In conclusion, four newly isolated K1-dependent phages (vB_EcoS_K1-ULINTec2, vB_EcoP_K1-ULINTec4, vB_EcoP_K1-ULINTec6, and vB_EcoP_K1-ULINTec7) demonstrated their efficacy in vitro by showing their ability to lyse K1 strains, even though none of them could be effectively active on all the strains. They were also able to increase the survival of *G. mellonella* larvae infected with *E. coli* K1, even if this did not result in a complete elimination of the inoculated bacteria.

## Figures and Tables

**Table 1:** Summary table of the groups of *G. mellonella* larvae inoculated for the survival and titration experiments.

	Groups	1st Injection	2nd Injection
1	APEC 134 + phage ^1^ MOI 100	APEC 134: 10^6^ CFU/10 µL	Phage ^1^: 10^8^ PFU/10 µL
2	APEC 134 + phage ^1^ MOI 10	APEC 134: 10^6^ CFU/10 µL	Phage ^1^: 10^7^ PFU/10 µL
3	APEC 134 + PBS	APEC 134: 10^6^ CFU/10 µL	PBS: 10 µL
4	PBS + phage ^1^ MOI 100	PBS: 10 µL	Phage ^1^: 10^8^ PFU/10 µL
5	PBS + PBS	PBS: 10 µL	PBS: 10 µL

^1^: vB_EcoS_K1-ULINTec2, vB_EcoP_K1-ULINTec4, vB_EcoP_K1-ULINTec6 or vB_EcoP_K1-ULINTec7. MOI: multiplicity of infection.

**Table 2:** Efficiency of plating (EoP) values for phages vB_EcoS_K1-ULINTec2, vB_EcoP_K1-ULINTec4, vB_EcoP_K1-ULINTec6, and vB_EcoP_K1-ULINTec7.

		Phages			
		vB_EcoS_K1-ULINTec2	vB_EcoP_K1-ULINTec4	vB_EcoP_K1-ULINTec6	vB_EcoP_K1-ULINTec7
***E. coli* strains**	45 *	1.000	1.000	1.000	1.000
	30 *	0.971	0.886	1.103	0.838
	79 *	NP	NP	/	NP
	134 *	0.765	1.143	1.205	2.137
	161 *	0.535	<0.001	<0.001	NP
	486 *	1.059	NP	NP	NP
	591 *	NP	0.829	0.692	0.906
	592 *	NP	1.000	0.405	1.086
	1073 *	NP	0.120	0.024	0.044
	1343 *	NP	NP	0.641	NP
	151.1 **	/	/	/	1.145
	143.1 *	NP	1.143	0.487	0.350
	O1 *	NP	0.343	NP	0.256
	O2 *	NP	0.629	/	0.197
	O7 *	NP	0.943	1.923	0.855
	O10 **	/	/	/	0.940
	O12 **	/	/	/	1.453
	O16 *	NP	0.005	NP	NP
	O45 *	NP	<0.001	0.564	2.821
	O166 *	NP	0.111	NP	NP

* K1 (*neuC*) positive strains, ** K5 (*kfib*) positive strains. EoP ≥ 0.5: high production (green); 0.5 ≥ EoP ≥ 0.1: medium production (yellow); 0.1 ≥ EoP ≥ 0.001: low production (red); EoP ≤ 0.001: no production (red); NP: no plaques.

## Data Availability

Sequencing was submitted as NCBI BioProject SUB10289878. GenBank accession numbers are MZ997838 (vB_EcoS_K1-ULINTec2), MZ997839 (vB_EcoP_K1-ULINTec4), MZ997840 (vB_EcoP_K1-ULINTec6), and MZ997841 (vB_EcoP_K1-ULINTec7).

## Supplementary Materials

The following are available online at https://www.mdpi.com/article/10.3390/v13102005/s1, Table S1: General characteristics of the *Escherichia coli* strains used in this study. Table S2: *Escherichia coli* serogroup collection and characteristics. Table S3: Primers used for PCR amplifications.
