# Supplementary material for: In Vitro Characterization and In Vivo Efficacy Assessment in Galleria mellonella Larvae of Newly Isolated Bacteriophages against Escherichia coli K1
Source: Viruses. 2021 Oct 6;13(10):2005. doi: 10.3390/v13102005 (PMC8541614; doi:10.3390/v13102005)
Supplement: Supplementary file 1 [file viruses-13-02005-s001.zip › viruses-1383720-supplementary.pdf]

viruses MDPI

Table S1. General characteristics of the *Escherichia coli* strains used in this study.

Pathotype	Origin	Reference	Serogroups and virulence factors
ExPEC			
	Avian	30	O18+, *neuC*+, *ibea*+
	Avian	45	O18+, *neuC*+, *ibea*+
	Avian	79	O18+, *neuC*+, *ibea*+
	Avian	134	O18+, *neuC*+, *ibea*+
	Avian	161	O18+, *neuC*+, *ibea*+
	Avian	486	O18+, *neuC*+, *ibea*+
	Avian	591	O18-, *neuC*+, *ibea*+
	Avian	592	O18-, *neuC*+, *ibea*+
	Avian	1073	O18+, *neuC*+, *ibea*+
	Avian	1343	O18+, *neuC*+, *ibea*+
UPEC			
	Human	15	*neuC*-
	Human	142.1	*neuC*-
	Human	143.1	*neuC*+
	Human	144.1	*neuC*-
	Human	144.2	*neuC*-
	Human	151.1	*neuC*-, *kfib*+ (K5)
	Human	154.2	*neuC*-
	Human	J96	*neuC*-
EHEC			
	Human	ATCC35150	O157+, H7+, *eae*+, *stx1*+, *stx2*+
	Bovine	40-498-8	O26+, *eae*+, *stx1*+, *stx2*-
EPEC			
	Human	E2348/69	O127+, *eae*+
Other	Human	ATCC25922	

Table S2. *Escherichia coli* serogroup collection and characteristics.

Serogroup	*neuC* (K1)	*Kfib* (K5)
O1	+	-
O2	+	-
O3	-	
O4	-	
O5	-	
O6	-	
O7	+	-
O8	-	
O9	-	
O10	-	+
O11	-	
O12	-	+
O13	-	
O14	-	
O15	-	
O16	-	
O17	-	
O18	-	
O19	-	
O20	-	
O21	-	
O22	-	
O23	-	
O24	-	
O25	-	
O26	-	
O27	-	

O28	-	
O29	-	
O32	-	
O33	-	
O35	-	
O36	-	
O37	-	
O38	-	
O39	-	
O40	-	
O41	-	
O42	-	
O43	-	
O44	-	
O45	+	-
O46	-	
O48	-	
O49	-	
O50	-	
O51	-	
O52	-	
O53	-	
O54	-	
O55	-	
O56	-	
O57	-	
O58	-	
O59	-	
O60	-	
O61	-	
O62	-	
O63	-	
O64	-	
O65	-	
O66	-	
O68	-	
O69	-	
O70	-	
O71	-	
O73	-	
O74	-	
O75	-	
O76	-	
O77	-	
O78	-	
O79	-	
O80	-	
O81	-	
O82	-	
O83	-	
O84	-	
O85	-	
O86	-	
O88	-	
O89	-	
O90	-	
O91	-	
O92	-	
O93	-	
O95	-	
O96	-	
O97	-	
O98	-	
O99	-	
O100	-	

O101	-	
O102	-	
O103	-	
O104	-	
O105	-	
O106	-	
O107	-	
O108	-	
O109	-	
O110	-	
O111	-	
O112AB	-	
O112AC	-	
O113	-	
O114	-	
O115	-	
O116	-	
O117	-	
O118	-	
O119	-	
O120	-	
O121	-	
O123	-	
O124	-	
O125	-	
O126	-	
O127	-	
O128	-	
O129	-	
O130	-	
O131	-	
O132	-	
O134	-	
O135	-	
O136	-	
O137	-	
O138	-	
O139	-	
O140	-	
O141	-	
O142	-	
O143	-	
O144	-	
O145	-	
O146	-	
O147	-	
O148	-	
O149	-	
O150	-	
O151	-	
O152	-	
O153	-	
O154	-	
O155	-	
O156	-	
O157	-	
O158	-	
O159	-	
O160	-	
O161	-	
O162	-	
O163	-	
O164	-	
O165	-	
O166	+	-

O167	-
O168	-
O169	-
O170	-
O171	-
O172	-
O173	-
O174	-
O175	-
O176	-
O177	-
O178	-
O179	-
O180	-
O181	-
O182	-
O183	-
O184	-
O185	-
O186	-
O187	-
O188	-

Table S3. Primers used for PCR amplifications.

Gene	Primers	Primer sequence (5'-3')	Size of PCR product (bp)	Annealing temp (°C)	Reference
neuC	neu1	AGGTGAAAAGCCTGGTAGTGTG	676	61	Moulin *et al.*, 2006
	neu2	GGTGGTACATCCCGGGATGTC			
kfib	kfib_F	AATTGTTTTAAAATCTGTTCT	501	52.5	Bekal *et al.*, 2003
	kfib_R	TGAGACTGAAATTACATTTAA			
wzx (O18)	OgC1-F	GTTCGGTGGTTGGATTACAGTTAG	551	58	Iguchi *et al.*, 2015
	OgC1-R	CTACTATCATCCTCACTGACCACG			
ibeA	ibeA_F	TGAACGTTTCGGTTGTTTTG	814	55	Germon *et al.*,2005
	ibeA_R	TGTTCAAATCCTGGCTGGAA			
